# Zn-containing Adhesives Facilitate Collagen Protection and Remineralization at the Resin-Dentin Interface: A Narrative Review

**DOI:** 10.3390/polym14030642

**Published:** 2022-02-08

**Authors:** Manuel Toledano, Manuel Toledano-Osorio, Matthias Hannig, Álvaro Carrasco-Carmona, María T. Osorio, Franklin García-Godoy, Inmaculada Cabello, Raquel Osorio

**Affiliations:** 1 Faculty of Dentistry, Colegio Máximo de Cartuja s/n, University of Granada, 18017 Granada, Spain; toledano@ugr.es (M.T.); alcarcar94@gmail.com (Á.C.-C.); rosorio@ugr.es (R.O.); 2 Clinic of Operative Dentistry, Periodontology and Preventive Dentistry, University Hospital, Saarland University, Building 73, 66421 Homburg/Saar, Germany; matthias.hannig@uks.eu; 3 Independent Researcher, 18014 Granada, Spain; mtoleosorio@gmail.com; 4 Health Science Center, College of Dentistry, University of Tennessee, 875 Union Avenue, Memphis, TN 381632110, USA; fgarciagodoy@gmail.com; 5 Integral Pediatric Dentistry Teaching Unit, Faculty of Medicine, University of Murcia, 30008 Murcia, Spain; icabello@um.es; 6 Murcian Institute of Biosanitary Research (IMIB), 30120 Murcia, Spain

**Keywords:** zinc, adhesive, polymers, dentin, remineralization, degradation, interface

## Abstract

This is a narrative review of the literature assessing the potential effectiveness of doping dentin polymeric adhesives with zinc compounds in order to improve bonding efficacy, remineralization and protection against degradation. A literature search was conducted using electronic databases, such as PubMed, MEDLINE, DIMDI and Web of Science. Through our search, we found literature demonstrating that Zn-doped dentin adhesives promote protection and remineralization of the resin-dentin interfaces. The increased bioactivity has also facilitated dentinal tubules’ occlusion by crystals’ precipitation contributing to improved sealing efficacy of restorations. Loading dentin adhesives with zinc gives rise to an increase of both crystallinity of mineral and crosslinking of collagen. The main role of zinc, in dentin adhesives, is to inhibit collagen proteolysis. We concluded that zinc exerts a protective effect through binding at the collagen-sensitive cleavage sites of matrix-metalloproteinases (MMPs), contributing to dentin matrix stabilization. Zinc may not only act as a MMPs inhibitor, but also influence signaling pathways and stimulate metabolic effects in dentin mineralization and remineralization processes. Zn-doped adhesives increase the longevity of dentin bonding through MMPs inhibition. Zn poses a remineralization strategy in demineralized dentin.

## 1. Introduction

**Figure 1:**
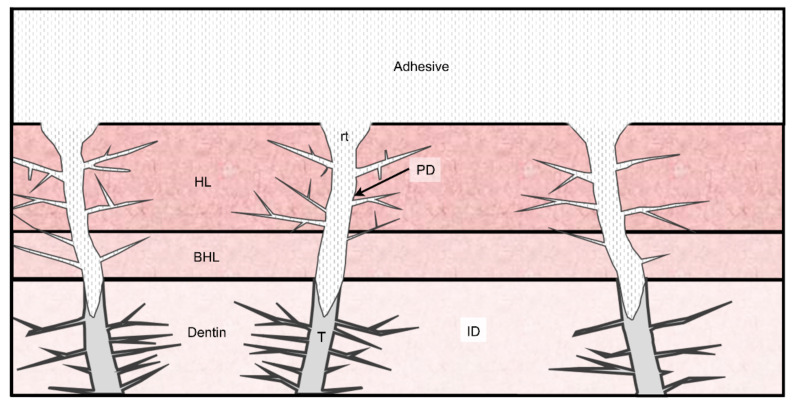
Resin-dentin interface schematic illustration. ID, intertubular dentin; PD, peritubular dentin; t, dentinal tube; rt, resin tag; HL, hybrid layer; and BHL, bottom of the hybrid layer.

The creation of dentin bonding is attained by two main strategies, based on the use of etch-and-rinse adhesives or self-etching adhesives. By using both techniques, a resin monomer diffusion decreasing gradient in the acid-etched dentin at the bottom of the hybrid layer results in a demineralized collagen matrix phase [1,2] (Figure 1). This zone is particularly susceptible to the proteolytic activity of host-derived or endogenous enzymes such as matrix metalloproteinases (MMPs) [3] found in the dentin [4,5,6]. In contrast to self-etching adhesives-treated dentin, MMPs are responsible for a higher collagen cleavage in phosphoric acid (PA)-etched dentin. However, an inhibition of collagen degradation in the hybrid layer [7] has been observed due to the presence of hydrophobic/hydrophilic resin monomers, resulting in a higher promotion of protection by the self-etch bonding approach [8].

A common practice in dentistry is bonding with adhesives to irregular dentin substrates, such as carious dentin. During clinical treatments, caries-affected dentin has to be preserved as it is able to be remineralized, acting as a viable dentin adhesion substrate [9]. Finally, demineralization can also be caused by a bacterial acid attack or by acidic food and beverages [10]. Bacterially derived enzymes can result in the degradation of exposed demineralized collagen, which is left without protection by the intrafibrillar minerals. As mineral deposition can fossilize activated MMPs and promote further nucleation, mineral reincorporation into the demineralized matrix of the dentin is determinant [11], as it causes inactivation of MMPs and allows unimpeded remineralization. Alternatively, the structural integrity of the collagen fibrils may be damaged by acidic conditioning. In order to facilitate a long-lasting adhesion, dentin adhesives should remineralize and protect the resin-dentin interfaces. The release of active molecules should trigger the bioactive nature of dentin [12]. Dentin remineralization is the restitution process of the inorganic matrix, which is clinically applicable for protection of the resin-dentin interdiffusion zone [13], against dentin caries and hypersensitivity, and so as preventive strategy [14,15]. Among the dentin sites requiring remineralization strategies of the exposed collagen fibrils are root caries, cervical erosion, and caries-affected dentin [10]. Physiologically, this process takes place on demineralized dentin surfaces. In these surfaces, damaged crystallites are rebuilt, and minerals are reabsorbed. The dentin remineralization dynamics is characterized by a high complexity.

Kim et al. [10] stated that the promotion of the growth nuclei centers, in dentin remineralization, require an intact collagen structure besides the residual mineral crystals’ presence. Highly mobile diffusing structures with a negative charge are formed in a loosely packed way by prenucleation clusters. Chemical and structural frameworks for mineral deposition are provided by the dentin. Dentin is formed by approximately 18% (w/w) of organic content. Around 10% of this content corresponds to non-collagenous proteins, while the other 90% is collagen. Among the non-collagenous proteins, phosphoproteins represent the vast majority. The anionic properties of these phosphoproteins may be involved on biomineralization [16]. The removal of the soluble portion of the organic matrix, which contains phosphoproteins, by using different decalcifying agents [17], is a key part in the remineralization of the demineralized dentin [18,19]. In a similar way, insoluble phosphoproteins found in decalcified collagen, have been speculated to enable remineralization in vitro due to their role as nucleation sites for mineral deposition [16].

**Figure 2:**
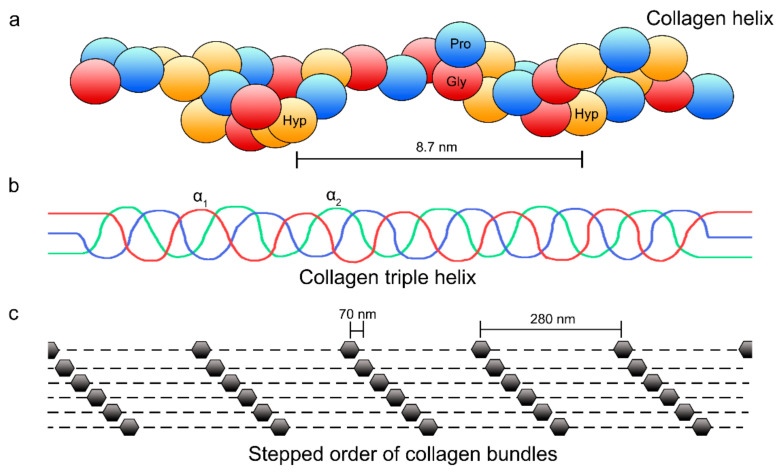
Each collagen helix (levorotatory strands referred as α chains) exhibits 3 residues of hydroxyproline (Hyp) (red marks), proline (Pro) (blue marks), and glycine (Gly) (yellow marks) sequences per turn (**a**). Three α chains form a quaternary structure that consists of a dextrorotatory helix (**b**). The collagen molecules gap zones are shown in (**c**), which unveil the typical staggered pattern of collagen fibrils due to the characteristic D- periodicity (67 nm).

At specific sites, dentin acts as a scaffold in the mineral deposition process [20]. At the gap border and overlap zones, the collagen fibrils exhibit nanosized positively charged regions. They can be used for mineral infiltration or charge–charge attractions [21,22], which in turn results in a higher degree of local energy [23] (Figure 2). Specific macromolecules are required in situ to act as crystal nucleators to regulate crystal size, stabilize the mineral phase and direct crystal orientation [24]. Partially demineralized dentin exhibits mineral-inductive properties due to the remaining polyanionic proteins, a small but definitive fraction that is firmly bound to the collagenous matrix [25]. Mineral induction is caused by a series of peptides that are found in the binding sites between the two collagen binding sites [20]. Therefore, the natural process of mineralization is imitated by the biomimetic mineralization, as it simulates the natural formation process of mineral crystals on organic and inorganic matrix surfaces without using aggressive conditions [26]. It has been observed that dentin plays an active role in tissue reparative processes, even in the absence of cells. The presence of growth factors, enzymes and bioactive molecules bound to the matrix that are liberated and activated through different mechanisms supports reparative processes [27]. Hence, nanometer-sized hydroxyapatite (HAP) grows and develops within nucleation sites [28]. This allows the formation of a collagen fibrils primary template, which enables their protection from enzymatic and hydrolytic degradation, partially reestablishing the tissue mechanical properties. The determination of these properties combines numerous factors, such as mineral location in the organic matrix, mineral density and microstructure [29].

**Figure 3:**
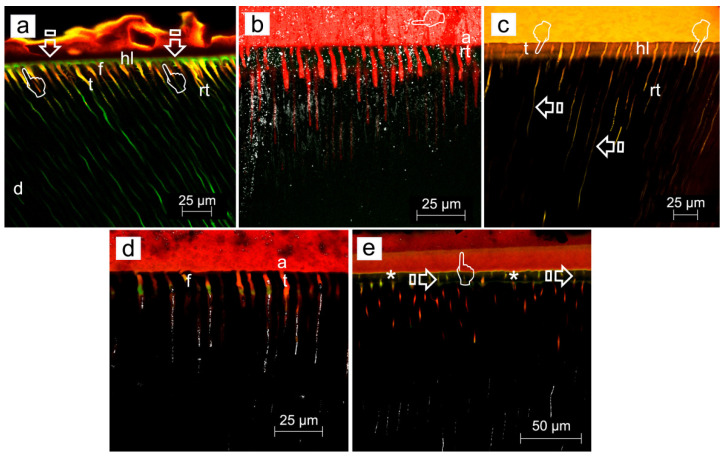
(**a**) Confocal laser scanning microscopy (CLSM) image (reflexion/fluorescence) of the resin-sound dentin interface created using phosphoric acid and Single Bond etch-and-rinse adhesive (PA+SB), after 24 h of storage, showing micropermeability (arrows) between dentin (**d**) and the adhesive layer (**a**). A strong spectral overlap (yellow) corresponds with patent signs of nanoleakage (pointers) at the hybrid layer (hl) (Scale bar: 25 µm). (**b**) A definitive lack of signs of nanoleakage is characterized, where both no penetration of fluoroscein throughout the proximal ends of dentinal tubules and water sorption in the thickness of the rhodamine B-labeled adhesive (**a**) can be observed when PA+SB-ZnCl_2_ is used (Scale bar: 25 µm). (**c**) CLSM image (reflexion/fluorescence) showing the interfacial characterization and micropermeability of the resin/caries-affected dentin interface created using phosphoric acid and Single Bond adhesive with dye xylenol orange (PA+SB·Xo) (Scale bar: 25 µm). (**d**) CLSM images (reflexion/fluorescence) screening the interfacial characterization and micropermeability of the resin/caries-affected dentin interface created by means of phosphoric acid and Single Bond adhesive ZnO-doped (PA+SB-ZnO) (Scale bar: 25 µm). (**e**) The interfacial characterization and micropermeability of the resin/caries-affected dentin interface created using PA+SB-ZnCl_2_ is shown at CLSM image (reflexion/fluorescence). A clear pattern of micropermeability within the dentinal tubules and the adhesive layer (arrows), and between the adhesive layer and the resin composite (pointer) may be pointed out (Scale bar: 50 µm). Previous to adhesive application, bond resins were doped with 0.05 wt% Rhodamine-B, or immersed in 0.5 wt% xylenol orange solution. Fluorescein was activated by blue light (488–495 nm) and emitted yellow/green (520 nm). The ultramorphology evaluation (resin-diffusion) was executed using Rhodamine excitation laser. Fluorescences were not separated into spectral regions to permit the operator a full control of the region of the light spectrum directed to each channel. More information about the used CLSM technique is provided at reference [31].

Collagenous hard tissues (i.e., bone and dentin) exhibit a mineral phase classified as either intrafibrillar apatite or extracellular apatite. The former is deposited immediately adjacent to or within collagen molecules gap zones and can be extended through the microfibrillar spaces in the fibrils, while the latter is found within the interstitial spaces that separate the collagen fibrils (Figure 2c and Figure 3). Intrafibrillar apatite seems to play a key role in the mechanical properties of the mineralized tissues [28,30].

It is well known that the presence of zinc preserves the dentin bonding effectiveness [14,32] and increases hard-tissue remineralization [33,34]. Zinc has been demonstrated to act not only as an inhibitor of matrix metalloproteinase (MMP) reducing MMPs-mediated collagen degradation [35] but also as a stimulator of hard-tissue mineral precipitation [36,37] and an inhibitor of dentin demineralization [38]. Zinc favors a metabolic effect in hard-tissue mineralization, influencing signaling pathways [32,37]. Zinc promotes the setting of a bond between material and tissue, eliciting a specific biological reaction at the interface [39]. Including zinc in the composition of the adhesives, intrafibrillar remineralization at the partially demineralized fibers will become augmented [9]. Zinc exerts both anti-inflammatory and antibacterial effects [40,41,42], as it decreases bacterial biofilm formation and growth [41]. This highlights the importance of Zn mineralized tissues [43].

The present study is a narrative review. It is a comprehensive summary that focuses on previously published scientific evidence. The aim of the present study was to conduct a review of the literature assessing the potential effectiveness of doping dentin adhesives with zinc compounds in order to improve bonding efficacy, e.g., facilitating collagen protection and remineralization at the resin-dentin interface.

## 2. Methods: Literature Search

A narrative exploratory review was undertaken. Literature search was conducted using electronic databases, such as PubMed, MEDLINE, DIMDI, Embase, Scopus and Web of Science. Hand-searching of the literature was also conducted, including the references lists of related and similar studies. The main search terms were “zinc” and “dentin” and “remineralization” and “adhesion” or “adhesives”. Only English-written articles were selected and no time limit was established. Articles designated and included in the present narrative review included those in which Zn-doped adhesives were studied in order to establish a possible application in the field of preventive and restorative dentistry. After thorough reading of the selected publications, performance of Zn-doped adhesives at the resin-dentin interface was evaluated considering bioactivity, remineralization, dentin collagen protection by means of carboxy terminal telopeptide of type I collagen (ICTP) determination, hybrid layer morphology, permeability, antibacterial properties and bonding efficacy.

## 3. Results

### 3.1. Zn-Doped Adhesives Effects on the Dentin Bonded Interface

**Figure 4:**
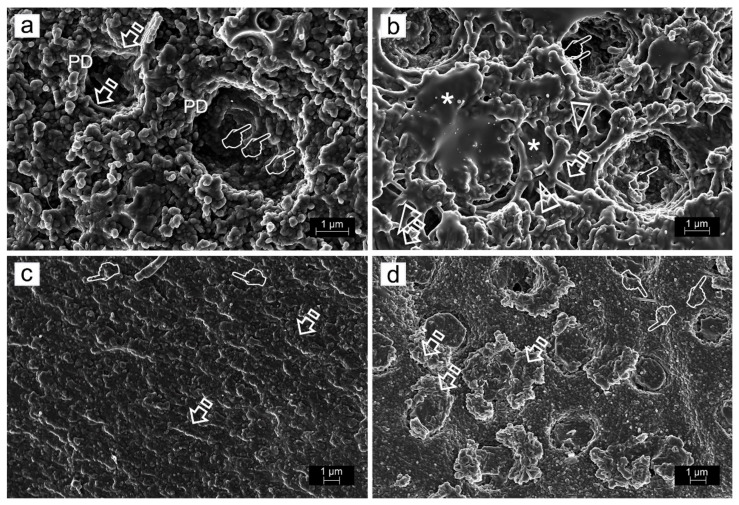
Field-emission scanning electron microscopy images of failures after bonding and microtensile bond strength testing. (**a**) The bonding component of the Clearfil SE Bond self-etching system doped with ZnO (SEB.Bd-ZnO) showed a mixed failure at the bottom of the hybrid complex. Tubules appeared mineral filled. (**b**) SEB.Bd-ZnO produced a mixed failure, affecting both the adhesive surface (asterisk) and the partially demineralized dentin (arrow) at the bottom of the hybrid complex. Mineralized dentin collagen without resin infiltration was detected inside some tubule wall, where crystals precipitated in knob-like formations (pointers). Multiple collagen fibrils appeared longitudinally mineralized (arrow heads). (**c**) The primer component of the Clearfil SE Bond self-etching system doped with ZnCl_2_ (SEB.P-ZnCl_2_) permitted to observe a mixed failure at the bottom of the hybrid complex. Multiple mineral formations are observed emerging through the resin–dentin infiltrated layer (arrows). (**d**) SEB.Bd-ZnCl_2_ originated a mixed failure at the top of the hybrid complex. A mineral deposition is completely covering the dentin surface. This substratum resulted totally mineralized and the mineral formations only allowed a restricted display of the tubule entrances (arrows). The typical staggered pattern of collagen fibrils due to the characteristic D- periodicity (67 nm) was visible at the fibers which cover the intertubular dentin (pointers).

**Figure 5:**
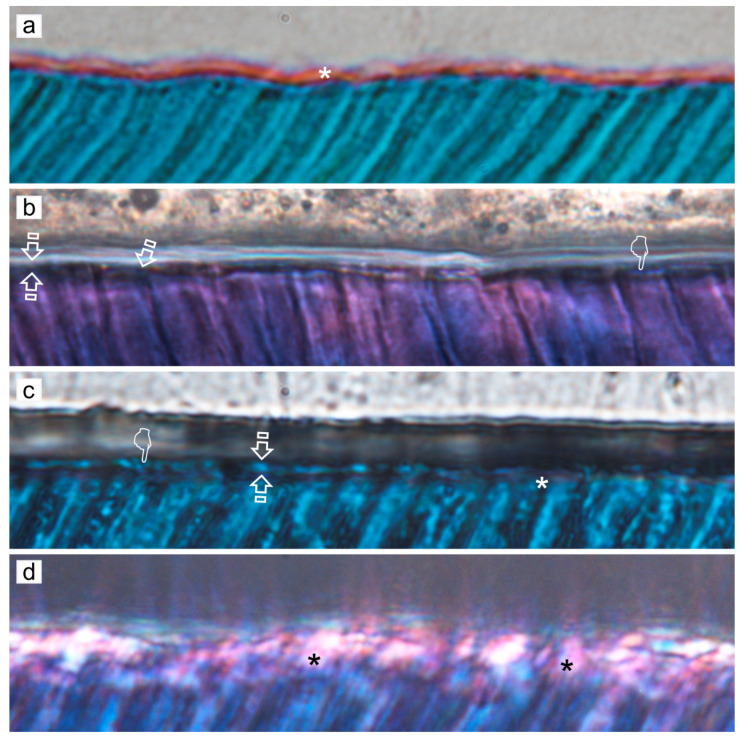
Representative light micrographs of SEB adhesive systems in sound dentin specimens; interfaces stained with Masson’s trichrome: mineralized dentin stained green, adhesive stained beige, and exposed protein stained red. Original magnification: 150×. (**a**) SEB control. (**b**) SEB.P-ZnO. (**c**) SEB.P-ZnCl_2_. (**d**) SEB.Bd-ZnO. Limited and clear resin uncovered decalcified dentin is shown (asterisk) (**a**). Evidence of partial demineralization or exposed protein may be detectable at the resin–dentin interface and tubular area (asterisks) (**c**). Slight and faint signs of demineralization show the scarce exposed proteins detected (arrows) (**b**). No signs of demineralization or exposed protein (red stain) are detectable at the resin–dentin interface when the primer was ZnO or ZnCl_2_ doped; clear observation of histological remineralization of the partially demineralized dentin layer is shown (pointer) (**b**). Absence of unprotected collagen layer is observable in some specimens (faced arrows) (**c**). Abbreviations: SEB, SE-Bond; SEB.P, SE-Bond primer; SEB.Bd, SE-Bond bonding; ZnO, zinc oxide; ZnCl_2_, zinc chloride.

**Figure 6:**
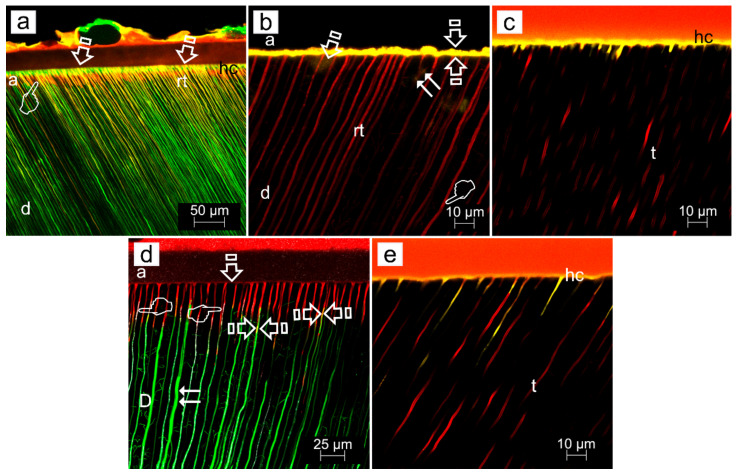
(**a**) CLSM image (reflexion/fluorescence) showing the interfacial characterization and micropermeability of the resin sound dentin-interfaces of SEB, after 24 h of storage, exhibiting micropermeability (arrows) between dentin (**d**) and the adhesive layer (**a**). A limited nanoleakage signal from the hybrid complex (pointer) (hc) may be observed. The adhesive layer showed thin resin tags (rt) when imaged in rhodamine excitation/emission mode (Scale bar: 50 µm). (**b**) CLSM single-projection image discloses the fluorescent calcium-chelators dye xylenol orange. It is observed the interfacial characterization of the resin/sound dentin interface created using SEB·P-ZnO and mechanical load cycling. Very little micropermeability and nanoleakage (arrow) between dentin (**d**) and the adhesive layer (**a**), and within the dentinal tubules (t) (double arrows), is shown. The presence of some obliterating mineral segment, is adverted (pointers). The adhesive layer displays an intense spectral overlap (yellow) (faced arrows). Long resin tags (rt) are also discernible (scale bar 10 µm). (**c**) CLSM single-projection images disclose the fluorescent calcium-chelators dye xylenol orange. The interfacial characterization of the resin/caries-affected dentin interface created using SEB·P-ZnCl_2_ adhesive applied on smear layer-covered dentin, and then load cycled, imaged in Rhodamine excitation/emission and calcium-chelators dye xylenol orange modes (SEB·P-ZnCl_2_·Xo,) is shown. The interface discloses a clear fluorescence signal within the hybrid complex (hc) and dentinal tubules (t) (Scale bar: 10 µm). (**d**) CLSM image (reflexion/fluorescence) of the interfacial characterization and micropermeability of the resin/carious-affected dentin-interface of SEB·P-ZnO and mechanically load cycled. Any micropermeability and nanoleakage (arrow) between dentin (D) and the adhesive layer (**a**), but within the dentinal tubules (t) (double arrows), is shown. No further dye diffuses into the adhesive layer and a powerful reflective signal within the dentinal tubules (pointers) is observed. Some resin tags show an intense spectral overlap (yellow) (faced arrows) (Scale bar: 25 µm). (**e**) CLSM single-projection image discloses fluorescent calcium-chelators dye xylenol orange. The interfacial characterization of the resin/caries-affected dentin interface created using SEB·Bd-ZnCl_2_ adhesive applied on smear layer-covered dentin, and then load cycled, imaged in Rhodamine excitation/emission and calcium-chelators dye xylenol orange modes (SEB·Bd-ZnCl_2_·Xo) is shown. The interface exhibits a clear fluorescence signal inside the hybrid complex (hc) and dentinal tubules (t) (Scale bar: 10 µm). All figures were obtained at 63x-2 with optical zoom at 10 µm of scale bar. a, adhesive layer; d, dentin; hc: hybrid complex; rt, resin tags; t, dentinal tubules. Previous to adhesive application, bond resins were doped with 0.05 wt% Rhodamine-B, or immersed in 0.5 wt% xylenol orange solution. Fluorescein was activated by blue light (488–495 nm) and emitted yellow/green (520 nm). The ultramorphology evaluation (resin-diffusion) was executed using Rhodamine excitation laser. Fluorescences were or not separated into spectral regions to permit the operator a full control of the region of the light spectrum directed to each channel. More information about the used CLSM technique is provided at reference [31].

ZnO doping of dentinal adhesives has been related to an improvement of the dentin remineralization and sealing efficacy, as clear signs of dentin remineralization, in etch-and-rinse adhesive procedures, protecting the hybrid layer integrity [31] (Figure 3a,c,d). When combined with self-etching adhesives, some calcium complexes were adverted at the resin-dentin interface [44] (Figure 4a,b, Figure 5a,b,d and Figure 6a,b,d). ZnO has also provided activity when doped into adhesive resins [40] without exerting cytotoxic effects on pulp fibroblasts [45].

**Figure 7:**
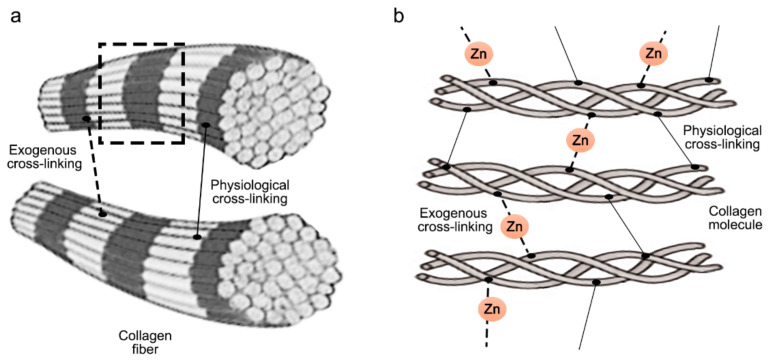
Scheme illustrating exogenous and physiological crosslinking induced as inter and intramolecular crosslinks. (**a**) crosslink between collagen fibrils, (**b**) dashed line area observed in a in more detail displaying individual collagen molecules composed of single α chain crosslinked by additional exogenous crosslinks (Zn).

When etch-and-rinse adhesives were doped with ZnCl_2_, collagen fibrils denoted an increase in crosslinking (Figure 7) and the attained sealing ability at the interface was improved, although it was not complete [31]. Clustered nodules of ZnCl_2_ were shown (pointer), and longer and strong resin tags (rt) were also observed (Figure 3b,e). ZnCl_2_ and ZnO in contact with partially demineralized dentin gave rise to mineral crystallinity [31]. When compared with ZnO, the dissolution rate of ZnCl_2_ was very fast [46], resulting in greater supersaturation of zinc phosphate [47].

Zn-doping promoted an improved sealing of the resin-dentin interface, a decrease of the hybrid layer porosity and an increase of dentin mineralization. Load cycling augmented the sealing of the Zn-doped resin-dentin interfaces, as porosity and nanoleakage diminished, and even disappeared in caries-affected dentin substrata conditioned with EDTA. Micropermeability at the resin-dentin interface diminished after combining EDTA pretreatment [48], ZnCl_2_-doping and mechanical loading stimuli on restorations. Severe micropermeability and water sorption were shown (arrows) in caries-affected dentin treated with PA+SB. A clear nanoleakage signal from the hybrid layer (pointers) placed underneath the rhodamine B-labeled adhesive layer could be observed. A substantial number of wide and long resin tags (rt) below the adhesive layer were present. Some resin tags showed an intense spectral overlap (yellow). Regular presence of xylenol-stained Ca-deposits within the hybrid layer (hl), walls of dentinal tubules (t) and resin tags (rt) was unveiled (Figure 3c). When caries-affected dentin was treated with PA+SB-ZnO, funneling (f) of the tubular orifices was discernible, with good penetration of the adhesive (a) into the entrance of tubules (t) (Figure 3d). In addition, a partial porosity and concise nanoleakage signals from the hybrid layer (asterisk) were observable when PA+SB-ZnCl_2_ was used in caries-affected dentin. The promoted new mineral formations contributed to reduce or avoid both porosity and nanoleakage from the load-cycled, Zn-doped resin-dentin interfaces. EDTA+SB-ZnCl_2_ (Single Bond doped with ZnCl_2_, previously etching dentin with EDTA) or SEB·Bd-Zn (bonding component of Clearfil SE Bond doped with Zn) are the preferred bonding procedures to treat caries-affected dentin surfaces. When SEB·Bd-ZnO was used, clumps of precipitation of minerals, in strata (pointers), covered the whole surface of dentin matrix, allowing the visual observation of tubules. Collagen fibers were clearly observed at the peritubular (PD), appearing totally mineralized (arrows) (Figure 4a). Nevertheless, at both intertubular and peritubular dentin, terminal knob-like structures were exhibited within the collagen fibers with SEB·P-ZnCl_2_. Tubule entrances appeared visible but completely precipitated by minerals (pointer) (Figure 4c). However, ZnO-doping is encouraged for etch-and-rinse adhesives in sound and caries-affected dentin [31].

### 3.2. Matrix Metalloproteinases and Collagen Degradation. The Role of Zn as MMPs Inhibitor

Zinc ions reduced collagen degradation in demineralized dentin through MMPs’ competitive inhibition [49]. Dentin collagen degradation was strongly reduced in demineralized dentin by high zinc concentration (3.33 mg/ml of zinc chloride solution) and this effect was maintained at least up to 3 weeks, as a clear sign of dentin remineralization [49]. MMP-mediated collagen degradation can also be inhibited by hydrophilic monomers through adsorption to the collagen fibrils [50]. Collagen degradation attained after pretreatment with zinc was four times lower than that obtained when using chlorhexidine in the same experiment [49]. MMP-mediated collagen degradation occurred in acid-etched and self-etched (SE)-primed dentin. Resin infiltration also decreased collagen degradation. Lower collagen degradation was found for SE Bond than for Single Bond. Zinc-doped Single Bond resin always reduced collagen degradation, the ZnO particles being more effective than ZnCl_2_. Zinc-doped SE Bond diminished the liberation of C-terminal telopeptide (ICTP) only at 24 h. Bond strength to dentin was not decreased when Zn-doped resins were employed, except when ZnCl_2_ was added to SE Primer. Zinc-doped resin reduced collagen degradation in Single Bond hybrid layers, but did not affect bond strength. The addition of zinc to SE Bond had no beneficial effects [35].

### 3.3. Measuring ICTP as the Most Reliable Indicator of MMP-Driven Collagenolysis in Dentin

Dentin matrix metalloproteinases are implicated in the pathogenesis of caries, contributing to collagen degradation in resin-dentin interfaces. Thereby, it has been proposed to determine if collagen degradation may be modulated by an excess of zinc or zinc chelators in mineralized and phosphoric acid demineralized human dentin specimens. Extremely low ICTP values have been obtained in experimentally mineralized bonded interfaces [49]. Mean ICTP values were affected by the conditioning media, dentin demineralization and storage time. Mineralized dentin samples released only negligible amounts of ICTP: 0.87 mg/L (24 h) to 4.04 mg/L (3 weeks), 0.39 to 1.8% of collagen degradation, respectively. When MMP-2 was added to the media, ICTP values increased significantly and ranged from 4.46 mg/L (24 h) to 9.02 mg/L (3 weeks), 1.98% to 4.01% of collagen degradation [49]. The amount of collagen degradation was significantly higher in phosphoric acid (PA)-demineralized dentin. The total amounts of ICTP liberated after incubation of PA-demineralized dentin in artificial saliva ranged from 70 mg/L (24 h) to 178 mg/L (3 weeks); these values increased significantly over time and corresponded to 31% and 79% of collagen degradation, respectively. ICTP values were lower at 24 h, and a significant increase in collagen degradation was observed after 1 week of incubation. These values were maintained significantly similar at the 3-- week evaluation period. When zinc was added, collagen degradation in demineralized dentin was reduced to 24% at 24 h, 53% after 1 week, and 60% after 3 weeks [49].

### 3.4. Dentin Remineralization. Zn-Substituted Apatite Compounds

It was questioned whether zinc may improve the repair ability of demineralized dentin [51]. For this purpose, dentin disks were demineralized by phosphoric acid during 15 s and immersed in artificial saliva, remineralizing solution, a zinc chloride solution and a zinc oxide solution, and they were analyzed after 24 h and 1 month of storage [51]. After phosphoric acid application, dentin is only partially demineralized. The CLSM images show a strong nanoleakage signal from the hybrid layer (pointers), when resin-sound dentin interface was created using phosphoric acid and just Single Bond etch-and-rinse adhesive (PA+SB), after 24 h of storage (Figure 3a). In this undoped sample, funnelling (f) of the tubular orifices was observable, with good saturation of the adhesive (a) into the entrance of tubules (t). The adhesive layer was characterized by long resin tags (rt). Demineralized dentin was remineralized after 24 h of storage (nanohardness increased and hydroxylapatite formation was detected by Raman) [51]. Remineralization was maintained up to 1 month in dentin stored in remineralizing solution, zinc chloride and zinc oxide. Zinc and phosphate are important in hydroxylapatite homeostasis. Scholzite (Zn-modified hydroxylapatite, CaZn_2_(PO_4_)_2_·2H_2_O) formation was encountered in dentin stored in zinc-containing solutions. Zinc might allow balance to be reached between dentin demineralization and remineralization processes [51]. In Zn-doped adhesive-dentin interfaces, a calcium phosphate layer and tubular occlusion was encountered at the debonded interface [52]. Under the stereomicroscope, crystal formations were found in both ZnO and ZnCl_2_-doped resin adhesives disks after 7 d of immersion in simulated body fluid solution. It was, detected by energy-dispersive X-ray spectroscopy (EDX), that the ZnO-doped resin produced Zn, Ca and P deposition (globular formations were observed by field emission scanning electron microscopy FESEM) after 7 d. Zn and P crystals were perceived by FESEM and EDX in the experimental ZnCl_2_-doped resin, after 7 d and 21 d. Hopeite (hydrated zinc phosphate, Zn_3_(PO_4_)_2_**·**4H_2_O)) formation was identified by Raman on both Zn-doped resins. Single Bond dentin adhesive did not produce mineral or crystal precipitation [47]. Zn homeostasis was regulated through Zn transporters, permeable channels, and metallothioneins (as histidine and cysteine, which are rich in thiols, causing them to bind a number of trace metals; e.g., metallothionein binds several Zn ions) [53].

## 4. Discussion

### 4.1. Zn-Doped Adhesives Effects on the Dentin Bonded Interface

Zinc has been widely applied in dentistry, as in root-canal filling materials, temporary fillings, cavity liners, filling materials, cementing media, oral rinsing solutions, denture-retention adhesives or toothpaste components. Zinc oxide cements release relevant amounts of soluble zinc ions, which usually exhibit higher solubility in humid environments [54]. At the resin/dentin interface, the activity of host-derived dentin proteases causes enzymatic degradation of the improperly impregnated dentin collagen [55]. Therefore, degradation of the hybrid layer can be avoided with the help of MMP inhibitors such as zinc. Zinc can also be applied to increase dental remineralization [51] by promoting bioactivity [47,56,57,58]. Bioactivity has been described as a characteristic property effect that produces a concrete biological response at the material’s interface, resulting in calcium and phosphate deposits [39]. Adhesives doped with zinc may be obtained with 20 wt% ZnO or 2 wt% ZnCl_2_ without altering the adhesive’s mechanical, chemical and physical properties [14,59,60].

ZnO exhibits basic properties and is classified as an amphoteric oxide. It is soluble and can be degraded in acidic conditions, while it is almost insoluble in alcohol and water. When ZnO is placed in dentin, it displays a preference for the demineralized dentin [52]. When ZnO contacts with acidic substrates, as some acidic non-collagenous proteins (dentin matrix proteins), higher solubility of ZnO is expected, and it could account for a more effective release of zinc ions [30,52] which stimulates protein phosphorylation, enhances calcium deposition [61] and facilitates partial dentinal tubules occlusion by crystals precipitation (Figure 4a,b). All this will alleviate or avoid hypersensitivity [62,63,64].

ZnO-doped adhesives liberate Zn^2+^ at a slower rate, which allows the formation of a ZnO-rich layer. This layer permits further mineralization and the formation of Ca and P deposits. When ZnO is combined with acids, its higher solubility permits zinc ion liberation in a more slow and effective way when the pH is lowered at the resin-dentin interface, as it happens in carious dentin [9]. Zinc might allow the balance between dentin demineralization and remineralization processes, performing as a new group of caries-preventive agents [65].

ZnCl_2_ is highly acidic [66], hydrophilic, and it has a soluble nature, thus it may produce an over-etching effect within the infiltrated dentin, when added to the adhesive constituents [67]. When ZnCl_2_ is applied in caries-affected dentin, it is able to further demineralize the underlying carious dentin, which has been previously demineralized [9,35,46]. This underlying dentin is over-demineralized due to the rapid diffusion of ZnCl_2_ throughout the substrate [46], probably increasing the local ions concentration, that will be available for further mineral growth (Figure 3d,e and Figure 4c,d). A thick hybrid layer was created when Single Bond (SB) adhesive was applied on PA-etched dentin, and the resin diffused within the porous dentin. In sound dentin, the interface presented funneled dentinal tubules, considered an essential sign of degradation of the poorly resin infiltrated demineralized and peritubular dentin. The excessive hydrophilicity of the resulted interface may have been the reason of the advanced resin degradation, as well as the extraction of water-soluble unreacted monomers or oligomers from the resin matrix. Discontinuities in the tubular filling with primer or adhesive are common, indicating the intermittent passage of fluorescein from the lateral tubuli toward the main dentinal tubules. Mechanical loading produced an outlined fluorescence which has been interpreted as the presence of consistent Ca-minerals deposited within the bonding interface and inside the dentinal tubules. When sound dentin was treated with EDTA+SB-ZnO, at the bottom of the hybrid layer, strong micropermeability between the Rhodamine B-labeled adhesive layer and dentin was adverted. Severe dye sorption throughout its thickness also appeared at the interface. Fluoroscein dye was accumulated at the entrance of some non-sealed tubules, though some other tubules were resin-filled. A poor reflective signal, i.e, scarce remineralization, was detected from the demineralized dentin layer and inside the dentinal tubules. On the contrary, when unloaded sound or carious specimens were treated with EDTA+SB-ZnCl_2_ less micropermeability between the dentin and the adhesive layer was generated, as points of overlapped light spectral (yellow) were observed. It complied with reduced signs of nanoleakage. Pronounced mineral components categorized this interface in sound dentin, as strong reflective signals from the inner dentinal tubules were also detected earlier. Load cycling applied on specimens treated with EDTA+SB-ZnCl_2_ did not produce signs of nanoleakage or water sorption. A moderate reflective signal from the demineralized dentin layer and inside the dentinal tubules was revealed. It indicated the presence of mineral components and no further dye diffused into the adhesive layer [31].

In combination with methacryloyloxydecyl dihydrogen phosphate (10-MDP)-based self-etching primers, Zn-10-MDP complexes are formed by the interaction between 10-MDPs and Zn^2+^ [68], which in turn may cause a reduction of the Ca-10-MDP salts formation [35] (Figure 4, Figure 5 and Figure 6). Simultaneous formation of MDP-Zn and Ca-MDP-Zn salts, rather than MDP-Ca, causes a compromise in free MDP penetration into the partially demineralized dentin [8,44,67,69]. The scarce MDP penetration is produced due to an obliterating and solid mineral segment presence [31,48]. Its nature is probably based on phosphate complexes, as 10-MDP, Zn^2+^ and Ca^2+^ reaction occurs during Zn-doped 10-MDP solution application on the dentin surface with a high concentration of calcium [69]. The formation of Zn-10-MDP complexes that reduces MDP dentin infiltration and the formation of Ca-MDP salts requires that Zn should be added into the bonding components for self-etching adhesives, but not in the primer [31]. Therefore, there is a higher preference for adhesive resins doped with ZnO than for those doped with ZnCl_2_ [32,59]. However, the use of materials that promote calcium and phosphate precipitation at the interface does not always guarantee the collagen remineralization [67]. SEB applied on smear layer-covered sound dentin produced a bonded dentin interface characterized by scarce micropermeability. Hence, limited both porosities and nanoleakage were observed. The adhesive layer was affected by reduced water sorption and put forward notable resin tags. Load cycling applied on these kind of specimens showed a conclusive lack of signs of nanoleakage. In addition, no further dye diffused into the adhesive layer and a robust reflective signal from the bottom of the hybrid complex and inside the dentinal tubules was detected. It means the existence of a firm and obliterating mineral segment [31].

### 4.2. Matrix Metalloproteinases and Collagen Degradation. The Role of Zn as MMPs Inhibitor

**Figure 8:**
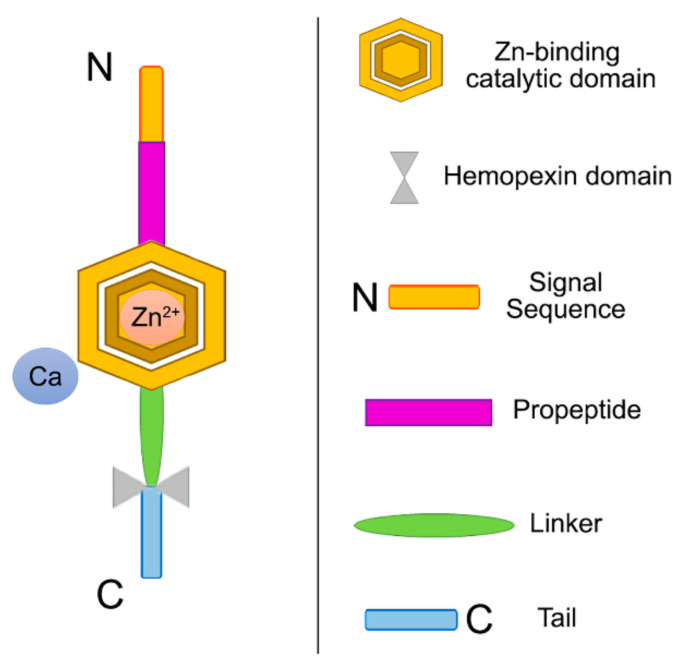
Schematic view of the modular domains of collagenases, a type of MMPs. MMPs are comprised of four domains; a signal peptide/sequence that is responsible of the protein secretion outside the cell; a propeptide region that is responsible for the inactivation of the enzyme until it is cleaved proteolytically; a catalytic domain with zinc and calcium where the propeptide region cysteine binds to inactivate it (cysteine switch); and a hemopexin-like domain that regulates substrate specificity and its interactions with endogenous inhibitors [68,71].

Among the multigene metzincin family are included several proteins, comprising meprins, bone morphogenic protein 1/tolloid-like metalloproteinase, transmembrane and secreted proteins called a disintegrin and metalloproteinases (ADAMs), and a disintegrin and metalloproteinase with thrombospondin motifs (ADAMTSs). MMPs are classified as members of this group [70]. Based on their cellular location, MMPs family is divided in two groups (secreted or membrane-bound), while their individual characteristics (determined by the substrate specificity or structure) allow a classification into six groups: membrane-type MMPs, matrilysins, stromelysins, gelatinases, collagenases, and others. Interstitial collagen I, II and III are primarily cleaved by collagenases. Their catalytic domains are involved in the cleavage of non-extracellular matrix (ECM) and non-collagen elements too. Their structure is composed of a prodomain, a catalytic domain, a hemopexin domain, and a transmembrane domain, glycosylphosphatidylinositol anchor or cytoplasmic tail at the end (Figure 8). The sequence PRCXXPD is highly conserved in all MMPs prodomains [70].

MMPs are typical endogenous dentin proteases widely distributed in oral saliva and dentin matrices [56,72,73], and are expressed as inactive pro-enzymes. The pro-domain interacting in the catalytic center with a zinc ion via a cysteine residue shields their catalytic domain. The removal of the pro-peptide causes the catalytic site to become accessible to substrates. A zinc ion coordinated between the three histidine residues located in the catalytic center mediates the endopeptidase activity. Proteolytic mechanisms involve an active site-bound water molecule polarization in order to act as a nucleophile to attack the scissile peptide bond polarized carbonyl group [74]. Anti-MMP drugs target catalytic zinc sites. This is triggered by the zinc ion, which can coordinate directly with many functional groups (i.e., hydroxyamates or sulphonamides), originating by a displacement of the zinc-water in the active site and an inhibition of the enzyme [75,76]. As stated above, the MMPs exhibit a catalytic zinc ion and a nonexchangeable one, which has been revealed to be a key part in the stabilization of the enzyme structure. MMPs also contribute to dentin mineralization. It is expected that reparative dentin calcification will be interfered with by the inhibition of these nonselective zinc/calcium chelators enzymes. The selectivity, side-effect potential and efficacy are not fully elucidated; however, it is clear that the leach of Zn^2+^ by dental materials (i.e., zinc oxide-eugenol cements, zinc phosphate cement, or silver amalgams) has been applied for decades with a high clinical success rate at dentin interfaces, allowing dentin remineralization while inhibiting in vivo dentin demineralization [76,77].

The exact inhibition mechanism of the enzymes mediated by zinc has been hypothesized previously [54] but not fully explained. Larsen and Auld [78] demonstrated that carboxypeptidase A is inhibited by zinc via zinc monohydroxide formation, bridging the catalytic zinc to an enzyme side chain in the active side. In proteins, zinc exhibits a structural role. In structural zinc sites, zinc ions are able to stabilize the proteins’ tertiary structure in an analogous way to disulphide bonds, with four amino acid side chains in a tetrahedral conformation. These tetradentate zinc complexes possess high stability constants [75]. The role of zinc in enzyme catalysis and protein folding/stability is also very important [79]. In procollagen and collagen molecules, four defined zinc-binding sites have been found, two located near each end and two at a distance of 126 and 206 nm from the C-terminal [80] near the MMPs fibronectin binding domain, amino acids 757–776 [81]. They show the same location as the sites for collagenase cleavage [80]. It has been suggested that zinc binding produces subtle conformational changes, leading to protection of the sensitive metalloproteinases cleavage sites. In other proteins, zinc binding produces N- or C-terminal modifications, inducing improvements in proteolytic resistance and thermostability [82].

The precise control of MMPs’ proteolytic activities takes place due to their precursors’ activation along with the inhibition of endogenous inhibitor proteins, tissue inhibitors of MMPs (TIMPs) and a-macroglobulin (a plasma protein that acts as a general inhibitor of proteinases) [83]. Brew and Nagase [84] literally stated: “The human genome contains four paralogous genes encoding TIMPs 1 to 4”. These four proteins from the TIMP family display a mainly similar structure [70]. While TIMPs were originally classified as MMPs inhibitors, recent investigations have established that they show a wide range of activities, such as the inhibition of ADAMTs, ADAMS, and disintegrin-metalloproteinases [70]. They attach and inhibit the MMPs activities at a molar stoichiometric proportion of 1:1, due to the creation of a N-domain composed of a special structure of 184 to 194 amino acids that inhibits MMPs’ catalytic activity, and a C-subdomain that mediates the interactions with the pro-MMPS hemopexin domain. MMPs do not generate covalent bonds with or cleavage TIMPs, but their proteolytic activity can be inhibited by the formation of tight complexes by TIMPs [70].

Hydroxyethylmethacrylate (HEMA) hydroxyl group coordination with zinc in the MMPs catalytic domain has been proposed as an inhibitory mechanism [50]. Reversible MMPs’ blocking can be caused by MMPs’ soluble adsorption to biomedical polymers (especially polymethylmethacrylate and poly-HEMA) [50,77,85]. The duration of this adsorption/blocking mechanism has yet to be clarified. Resin hydrolytic degradation and solubilization [86,87] can be involved in the cleavage of the unprotected collagen fibrils mediated by the MMPs within the decalcified dentin [7,56,76]. The creation of metal-activated switches for the increase of dentin collagen stability requires research on metallic ion binding sites on demineralized dentin.

### 4.3. Measuring ICTP as the Most Reliable Indicator of MMP-Driven Collagenolysis in Dentin

**Figure 9:**
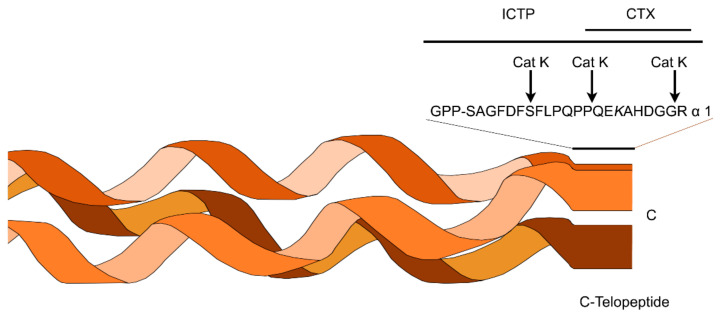
CTX and ICTP are epitopes that can be used as bone resorption markers on cathepsin K (Cat K) and type I collagen cleavage sites. In this schematic representation the structures of CTX and ICTP are represented. CTX epitope comprises an eight amino acid sequence on the α-1 C-telopeptide, while the ICTP one exhibits a larger conformation with two telopeptides and the first phenylalanine of the phenylalanine rich region Cathepsin K degrades the ICTP epitope, while generating CTX. The hydroxylysine (*K*) can act in inter- and intramolecular covalent crosslinks [88].

The ICTP is the carboxy terminal telopeptide of type I collagen, joined via trivalent crosslinks and liberated during collagen degradation. Through matrix metalloproteinases action, the telopeptide is produced, and it can be considered an MMP-driven collagenolysis index [88] (Figure 9). ICTP determination has been defined as a reliable analytical procedure for the quantification of the enzymatic activity on type I collagen [56,88,89,90,91]. In enzymatic assays with MMPs, the presence of native substrates (i.e., collagen) is recommended, when the tissue MMP inhibitor is present in physiologically relevant settings [90], but they produce experimental limitations. The assessment of specific MMPs’ kinetic studies may not be possible due to: (1) individual variabilities in MMPs expression/activity [80] and (2) the overlapping metzincin subfamilies substrate specificity; this makes quantification of individual proteinase activities impossible [74]. Zinc ions act directly on the bond-breaking procedure in the MMPs’ catalytic domain [75]. This highlights the importance of the zinc presence around bound MMPs for the determination of their bioactivity and mineral precipitation ability [57,58].

In other zinc-doped biomaterials, the calcium phosphate layer precipitation has been described, with a more solubility-resistant hydroxyapatite, for which solubility and quality is strictly dependent on zinc content and concentration [92]. Moreover, crystals’ precipitation caused by zinc favors tubular occlusion (Figure 4). Precipitated crystals are not easily dissolved after exposure to acids [93]. Therefore, the production of CaP precipitates induces the formation of high molecular-weight complexes (CaP:MMP) that have restricted mobility [94]. A prolonged exposure to hydroxyapatite (>4h) reduces ICTP values measurements and inactivates some MMPs [95]. Among the substrate specificities of MMP-2 and MMP-9, the type I collagen is included [43], while they are involved in the degradation of ECM components. MMPs’ release or union to the ECM and their subsequent activation cause their regulation by co-secreted or ECM-anchored endogenous TIMPs [96]. MMP-2, -8, -9, -20, located in the dentin, contribute to dentin matrix organization and mineralization, while also acting in the dentin matrix modulation during caries progression [97,98].

Tissue collagen concentration instead of its degradation [99] can be assessed by detection of collagen constituents, i.e., hydroxyproline [100]. Other collagen fragments (such as the C-telopeptide CTX epitope) may not be as specific and sensitive as ICTP [88]. Only MMPs, among known collagenolytic proteinases involved in the resorption of hard tissue, can generate ICTP. This liberation can be inhibited by MMPs’ inhibitors, while cysteine proteinase inhibitors are unable to do so [88]. Gelatin zymography has been applied for the determination of MMPs activity in dentin substrates [101,102], but the accuracy of the quantification with densitometry is not high and profiling is limited [74]. Moreover, the zymography technique does not distinguish between TIMP-inhibited and free enzymes [49].

### 4.4. Dentin Remineralization. Zn-Substituted Apatite Compounds

The process of remineralization includes the transformation of amorphous calcium phosphate (ACP) deposits to hydroxyapatite (HAP), minerals nucleation from prenucleation clusters, and postnucleation growth control [24]. The ACP [103] octocalcium phosphate [Ca_8_H_2_(PO4)_6_·5H_2_O] and tricalcium phosphate [Ca3(PO_4_)_2_] phases have also been identified as precursors [104]. Biological apatite exhibits a deficiency in the calcium concentration while carbonate is present in substantial amounts [105]. Another HAP precursor is the carbonated apatite. When zinc is used for remineralization, differences in Zn/Ca ratios may be the function of selectivity for a specific MMP [75,106]. Son et al. [107] concluded that, based on the crystals structure theory, the zinc fills the vacancy or the interstitial sites of crystal lattice, thereby presenting as a loss of certain electrical neutrality in an easy way when the doped ions (Zn: 0.074 nm) radii are smaller than Ca (0.099 nm). This creates more vacancies for point defects as an equivalent charged ion is lost. The stoichiometric formula for HAP is Ca_10_(PO_4_)_6_(OH)_2_. Despite this, it has been concluded that there is a calcium deficiency in biological apatite, while there are substantial amounts of carbonate [105]. Carbonated apatite is highly soluble, providing multiple ions for further remineralization. When zinc is present, carbonated apatite precipitates, triggering an exchange between Zn^2+^ and Ca^2+^ and forming a substituted apatite compound [108]. When Ca^2+^ is replaced by Zn^2+^, an isomorphous substitution into dentin HAP occurs [51]. Scholzite crystals -CaZn_2_(- PO_4_)_2_-2H_2_O- formation in dentin has been previously described [51]. When Ca-deficient apatite interacts with Ca^2+^ solutions, there is an improvement in stoichiometry and it could convert to HAP [109]. Stoichiometric HAP exhibits remarkable mechanical properties, low precipitation and dissolution rates, and the formation of crystals takes place for longer time periods [110].

The binding of collagen and other oligomeric proteins from the matrix is promoted by zinc. The interactions between proteins can be controlled by metallic cations and dictated by zinc ions as the preferential ligand [111]. When a binding model predicts that there is a single binding site on a protein, it indicates a preference for a specific cationic species, usually exhibiting a high affinity for them. On the other hand, when the protein molecule displays different binding sites, distinct cation species can be put up, each one with different affinities for the structure [111]. In these structures, amino-acid side chains that serve as ligands for zinc are able to create hydrogen bonds with other residues to allow a three-dimensional orientation of the binding-site configuration and a decrease of the metallic ion binding entropy [75]. Current data suggest that there is no particular binding model predilection; distinct divalent cations exhibit different affinity constants to collagen. In the catalytic zinc site, zinc-bound water is a key part in the zinc role, as a powerful electrophilic catalyst provides all or a combination of: (1) an activated water molecule for nucleophilic attack, (2) scissile bond carbonyl polarization, and (3) the negative charge in the transition state stabilization [75].

**Figure 10:**
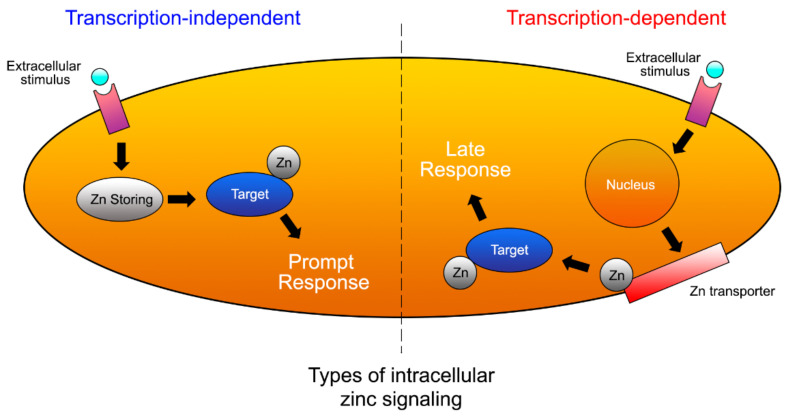
Schematic representation of Zn signaling at prompt and late stages. Zn levels in prompt signaling are directly increased by extracellular stimulus. Zn is released within minutes from a Zn store. Several hours after stimulation, late signaling is induced. Transcriptional changes in the expression of Zn transporters affect late Zn signaling [53].

Acid exposes the collagen scaffold to be remineralized, but it can also activate the dentin MMPs pro-forms. Effective MMPs inhibitors can protect the seed crystallite-sparse collagen fibrils of the scaffold from degradation before they can be re-mineralized [112]. In addition to its role as an MMP inhibitor, zinc can also stimulate metabolic effects and guides signaling pathways in hard-tissue mineralization [36] and remineralization processes [37] (Figure 10). It has also been observed that zinc not only inhibits dentin demineralization [38] but facilitates enamel remineralization [37]. Depending on the concentration of the divalent ions, Ca^2+^ can inhibit Zn^2+^ binding. The inhibitory effect of excess zinc ions on MMPs may be caused by the presence of Ca^2+^ in the local environment [80,106]. As in other proteins that require Zn^2+^ to bind to collagen, any local variation in the Ca^2+^ and Zn^2+^ mass ratio can result in a modulation of the interactions between MMPs and collagen. The differential Zn^2+^ hydration free-energy depends on its zinc-water coordination geometry. Zinc-water/protein interactions are able to decrease the Zn^2+^ hydration free-energy to the other divalent cations free energies (i.e., calcium) [79].

### 4.5. Limitations of the Present Research

Several limitations have been found when using zinc ions within the chemical formulation of the adhesives. The use of native substrate (i.e., collagen) in the presence of tissue MMP inhibitor, and in a physiologically relevant setting is recommended for performing enzymatic assays with MMPs, but reveal experimental limitations. Kinetic studies of specific MMPs may not be assessed due to: (1) the individual variability in MMP expression/activity and (2) the overlapping substrate specificity of metzincin subfamilies; thus, quantification of individual proteinase activity is not possible. Both points require further investigation. Additional research is also required, as it seems that selectivity for a specific MMP activity may also be related to differences in Zn^2+^/Ca^2+^ ratios. Ca^2+^and/or Zn^2+^ binding will occur depending on the relative abundance of these divalent ions. Local variations in the mass ratio between Ca^2+^ and Zn^2+^ may modulate metalloprotein-collagen interactions, as occurred with other different proteins able to bind collagen by Zn^2+^. The analysis of the limited literature available indicates that further specific studies on the relationship between the dentin microstructure and the physicochemical properties of zinc-doped adhesives should be determined. It remains to be ascertained whether zinc could have other local side effects on the connective tissue around the adhesive layer. Though the inclusion of zinc at the hybrid layer seems to facilitate mineral deposition on the demineralized dentin interface, this hypothesis needs further evaluation. It has been speculated that zinc-water/protein interactions may have decreased the hydration free-energy of Zn^2+^ relative to the free energies of other divalent cations such as calcium. This hypothesis of differential metallic ion-binding affinity is also indirectly supported by data available from other proteins such as metallothioneins. This lack of clarification may represent a further limitation. Raman analysis should also be performed, to clarify the appearance of new peaks in Amide III in presence of zinc. Long lasting and in vivo clinical studies are required, in order to determine whether these actions are durable at resin–dentin bonded interfaces. Additional limitations come from the unavailability of some techniques of research as transversal microradiography (TMR) or micro-X-ray diffractometry (μXRD2) and Transmission Electron Microscopy/selected area diffraction (TEM/SAED). These technologies should be able to contribute, in the near future, to open new insights in the zinc-dentin interaction. Therefore, these limitations may serve as future strategies of research, emphasizing on a double working field: in vivo clinical studies and systematic reviews about shortcomings of Zn-doped adhesives.

## 5. Conclusions

Based on the present review, we propose to encourage manufacturers and the pharmaceutical industry to include zinc ions in the synthesis of novel dentin adhesives. This proposal is based on the following key assumptions, (i) zinc ions, located in biological systems, are considered MMPs’ competitive inhibitors for collagen degradation [35]; (ii) when they bind to the MMPs’ collagen-sensitive cleavage sites, they exert a protective function, contributing to dentin matrix mineralization and organization [52]; (iii) collagen serves as an active scaffold in the oriented crystalline HAP formation within the fibrils [21,22]; (iv) zinc contributes to the increase of resin/dentin bonded interfaces’ longevity.

When zinc ions are present, the Raman analysis of the hydroxyproline exhibits a characteristic band corresponding to the protective effects of Zn derivatives on the structure stability of subfibrillar triple-helical collagen [113,114]. This indicates tissue collagen concentration [99], ideally for dentin remineralization and mineral precipitation. The high demand of engineered dental tissues has pointed out a requirement for new adhesive/primers formulations including zinc in their synthesis. Therefore, the need for the production of zinc-doped adhesives is rising. In recent years, zinc addition to resin-based dental adhesives has been researched. The inclusion of zinc methacrylate, zinc chloride or even oxide has proven to be an effective way for the doping with zinc [115], in the adhesive formulations [35]. The addition of this ion does not affect the strength of the dentin bond, but it is able to increase dentin bonds’ longevity through MMPs’ inhibition [35,47,115]. High concentrations of zinc could act as effective, stable and potent collagen degradation inhibitors mediated by dentin MMPs [49,115,116,117].

## Data Availability

The data presented in this study are available on request from the corresponding author.

## Abbreviations

ACP	Amorphous calcium phosphate
ADAM	A disintegrin and metalloproteinase
ADAMT	A disintegrin and metalloproteinase with thrombospondin motifs
CLSM	Confocal laser scanning microscopy
ECM	Extracellular matrix
EDX	Energy-dispersive X-ray spectroscopy
FESEM	Field Emission Scanning Electron Microscopy
HAP	Hydroxyapatite
HEMA	Hydroxyethylmethacrylate
ICTP	Carboxy terminal telopeptide of type I collagen
MDP	Methacryloyloxydecyl dihydrogen phosphate
MMPs	Matrix metalloproteinases
μXRD2	Micro-X-ray diffractometry
PA	Phosphoric acid
TEM/SAED	Transmission Electron Microscopy/selected area diffraction
TIMP	Tissue inhibitor of metalloproteinases
TMR	Transversal microradiography
